# Preparation and Investigation of Amphiphilic Block Copolymers/Fullerene Nanocomposites as Nanocarriers for Hydrophobic Drug

**DOI:** 10.3390/ma10020192

**Published:** 2017-02-16

**Authors:** Qinggang Tan, Yanyan Chu, Min Bie, Zihao Wang, Xiaoyan Xu

**Affiliations:** Key Laboratory for Advanced Civil Engineering Materials (Ministry of Education), School of Materials Science and Engineering, Tongji University, Caoan Road 4800, Shanghai 201804, China; 1433081@tongji.edu.cn (Y.C.); 1531505@tongji.edu.cn (M.B.); 1631415@tongji.edu.cn (Z.W.); kadxxy@tongji.edu.cn (X.X.)

**Keywords:** nanocarriers, nanocomposites, amphiphilic block copolymers, fullerene, controlled drug delivery

## Abstract

Biopolymer/inorganic material nanocomposites have attracted increasing interest as nanocarriers for delivering drugs owing to the combined advantages of both biopolymer and inorganic materials. Here, amphiphilic block copolymer/fullerene nanocomposites were prepared as nanocarriers for hydrophobic drug by incorporation of C60 in the core of methoxy polyethylene glycol-poly(d,l-lactic acid) (MPEG-PDLLA) micelles. The structure and morphology of MPEG-PDLLA/C60 nanocomposites were characterized using transmission electron microscopy, dynamic light scattering, high-resolution transmission electron microscopy, and thermal gravimetric analysis. It was found that the moderate amount of spherical C60 incorporated in the MPEG-PDLLA micelles may cause an increase in the molecular chain space of PDLLA segments in the vicinity of C60 and, thus, produce a larger cargo space to increase drug entrapment and accelerate the drug release from nanocomposites. Furthermore, sufficient additions of C60 perhaps resulted in an aggregation of C60 within the micelles that decreased the drug entrapment and produced a steric hindrance for DOX released from the nanocomposites. The results obtained provide fundamental insights into the understanding of the role of C60 in adjusting the drug loading and release of amphiphilic copolymer micelles and further demonstrate the future potential of the MPEG-PDLLA/C60 nanocomposites used as nanocarriers for controlled drug-delivery applications.

## 1. Introduction

Chemotherapy, i.e., the use of chemotherapeutic drugs to kill cancer cells, is one option and, sometimes, the only available to control tumor progression, improve quality of life, and extend survival in cancer patients [1,2,3]. However, the administration of chemotherapeutic drugs to tumor sites still remains a main challenge for efficient chemotherapy because of the poor bioavailablity of chemotherapeutic drugs caused by their poor solubility and in vivo instability as well as the rapid blood clearance of most chemotherapeutic drugs [4,5]. Nanoscale drug carriers provide potential to overcome these challenges in chemotherapeutic drug administration due to the advantages of nanoscale carriers used for drug delivery include the ability to enhance drug bioavailability, improve the blood circulation time, reduce systemic side effects caused by drugs, and enable delivery of drug molecules directly into the targeted cells [6,7,8,9].

Nanosized amphiphilic copolymer micelles have attracted much attention as nanoscale drug delivery carriers [10,11,12,13]. These drug delivery carriers consist of a hydrophobic inner core serving as a reservoir for hydrophobic chemotherapeutic drugs and a hydrophilic shell stabilizing the hydrophobic core and rendering polymer water-soluble. This core-shell structure characteristic enables the hydrophobic chemotherapeutic drugs efficiently entrap or encapsulate in the core, thus allowing for protection of drugs from hydrolytic or enzymatic degradation, reduction of drug clearance rates, improvement of the timed release of drug molecules in a sustained and continuous delivery manner, thereby reducing plasma fluctuation in circulating drug levels, as well as minimizing side-effects caused by chemotherapeutic drugs. The hydrophilic shell, in particular composed of the polyethylene glycol (PEG) segment, not only serves as a stabilizing interface between the hydrophobic core and the external medium, but can also escape rapid uptake by the phagocytic cells of the reticuloendothelial system (RES) and, thus, result in prolonged blood circulation times, which provide a potential for sustained and targeted delivery drug molecules to site of action [14,15]. Therefore, nanosized amphiphilic copolymer micelles have emerged as useful and effective chemotherapeutic drug carriers for therapeutic treatment in oncology.

Suitable chemotherapeutic drug loading capacity and drug release rate play a key role in amphiphilic block copolymer micelles as nanoscale carriers to achieve an effective chemotherapy for cancer [16]. Generally, the drug loading capacity of micelles formed from amphiphilic block copolymers is largely related with the interaction between the drug and the hydrophobic segment. The stronger interactions enable more hydrophobic chemotherapeutic drug to be trapped in the inner hydrophobic core of the micelles [17,18].

However, the release of drugs from block copolymer micelles will depend upon the rate of diffusion of the drug from the micelles, and the interaction between the drug and the core-forming block is stronger, a considerable decrease in the release of the drug from the micelle can be observed [19,20]. That the strong core-forming block–drug interactions will enhance loading and decrease the release rate of the drug from the micelles brings a challenge in the rational design of micelles as drug delivery systems to achieve the reasonable drug loading level and the desired release kinetics of the drug from micelles.

Due to its intrinsically appealing photochemical, electrochemical, and physical properties, fullerene (C60), a unique class of carbon allotropes, has been shown to be potentially useful as a nanoscale drug carrier [21,22,23,24,25]. Fullerene has a strong apolar character that can be useful as a drug absorbent. However, this apolar character also makes fullerene poorly soluble in physiological media and limit its use in biology. Generally, a suitable chemical modification for fullerene is designed to resolve their poor dispersion for biological applications [26,27]. It should be noted that the surface chemical modification may also cause damage to the physicochemical properties of fullerene.

Recently, biopolymer/inorganic material nanocomposites are attracting increasing interest as nanocarriers for delivering drugs owing to their unique structure and properties [28,29,30,31]. These nanocomposites can combine the advantages of both components (inorganic and biopolymer) and show some new and interesting properties used as nanocarriers for anticancer drug delivery. Herein, we report the methoxy polyethylene glycol-poly(d,l-lactic acid) (MPEG-PDLLA)/C60 nanocomposite micelles of a typical amphiphilic copolymer and fullerene as the nanocarriers for hydrophobic drugs, which combines the advantages of both amphiphilic block copolymer and fullerene delivery systems. In this constructed nanocomposite, the intact C60 may be dispersed into the hydrophobic core of amphiphilic copolymer micelles via the hydrophobic interaction and, thus, resolve the problem of dispersion of C60 in physiological media. In addition, owing to the uniquely nanoscale spherical structure, C60 incorporated into the hydrophobic segments of amphiphilic copolymer might have significant effects on the space structure of core of micelles and, therefore, on drug loading and delivery performance. A typical hydrophobic chemotherapeutic drug, doxorubicin, is used to investigate the drug loading and in vitro release behavior in phosphate buffer solution (PBS). The introduction of C60 to methoxy polyethylene glycol-poly(d,l-lactic acid) micelles could effectively adjust the drug loading and release behavior. The results obtained also provide fundamental insights into understanding of the C60 role to adjust the drug loading and release of amphiphilic polymer micelles.

## 2. Results and Discussion

**Figure 1:**
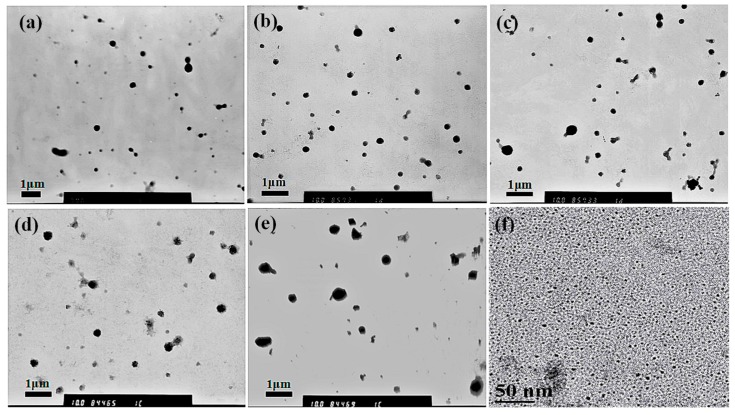
Transmission electron microscope (TEM) images of drug loaded MPEG-PDLLA/C60 nanocomposites prepared from different added amounts of C60 to the MPEG-PDLLA/C60 mixed solutions: 0% (**a**); 1% (**b**); 5% (**c**); 10% (**d**); 20% (**e**) and the high- resolution transmission electron microscopy (HR-TEM) images of drug-loaded C60 (**f**).

To explore the effects by only changing the added amount of C60 on the structure of MPEG-PDLLA/C60 nanocomposite, we prepared the nanocomposites by using MPEG-PDLLA at a fixed concentration of 1.5 mg/mL (above the critical micelle concentration to ensure the formation of polymer micelles), with varying added amounts of C60. The morphology of the resulting nanocomposites was studied by transmission electron microscope (TEM). Figure 1 shows the TEM micrograph of the MPEG-PDLLA/C60 nanocomposites. As shown in Figure 1a, in the absence of C60, the MPEG-PDLLA molecules self-assembled into micelles with well dispersed and appeared as individual nanoparticles with almost spherical shape in an average size of about 230 nm. The observed spherical structure from the TEM data confirmed a successful micellization of the MPEG-PDLLA block copolymers in water media. When the C60 was added, the self-assembled structures of MPEG-PDLLA/C60 nanocomposites also exhibited similar spherical structures with that of pure MPEG-PDLLA block copolymers as shown in Figure 1b–e. However, the average size of the spherical micelles increased with the increasing added amount of C60 to the MPEG-PDLLA/C60 mixed solution. Thus, it is apparent that C60 did not disturb the self-assembly behavior of MPEG-PDLLA block copolymers, but the C60 incorporated into the micelle could cause the micelles to increase in diameter size.

**Figure 2:**
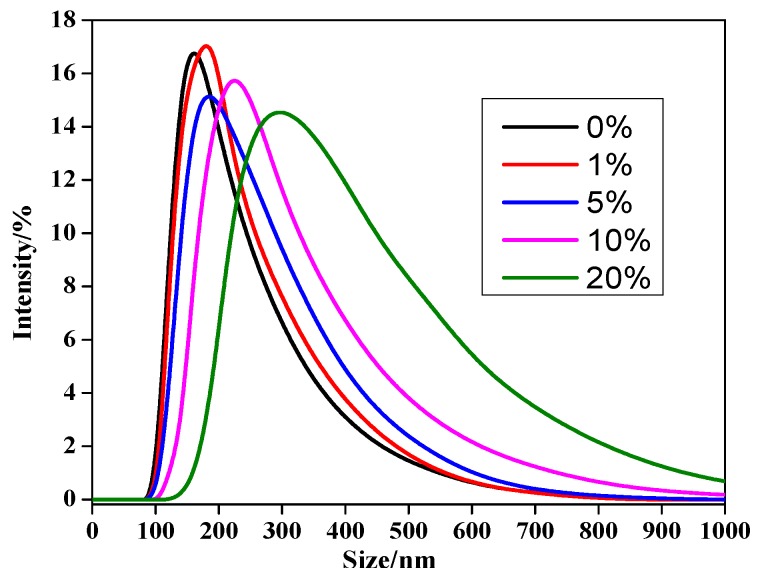
Size distributions of drug-loaded MPEG-/C60 nanocomposites prepared from different added amounts of C60 to the MPEG-PDLLA/C60 mixed solutions (determined by DLS at 25 °C).

To gain more intuitive data about the effect of C60 on the size of nanocomposite, the size and distribution of the MPEG-PDLLA/C60 nanocomposites in water were studied by dynamic light scattering (DLS). Figure 2 shows the variation of intensity-averaged diameter of the MPEG-PDLLA/C60 nanocomposites with different added amounts of C60. From the data obtained we can calculate that the intensity-averaged diameter of the polymer micelles at 25 °C was about 280 nm before addition of C60. Subsequently, the introduction of C60 also results in a similar increasing trend in hydrodynamic diameter as that observed by TEM.

The difference in diameters obtained from TEM images and DLS data were compared as shown in Table 1. As expected, the hydrodynamic diameter of the micelles determined by DLS is generally larger than what had been calculated from the TEM images (cf. Figure 1). This finding is most likely due to the fact that the shell-chain of the micelles completely extend in water while the micelles usually shrink during the progress of the sample drying for TEM analysis. Therefore, the TEM images and DLS data clearly indicated that the MPEG-PDLLA/C60 nanocomposites were successfully prepared by the membrane dialysis of the MPEG-PDLLA and C60 mixed solutions against water. As-obtained nanocomposites exhibited well-defined spherical structures with nanoscale size, representing crucial feature for the development of nanoscale drug carriers.

**Figure 3:**
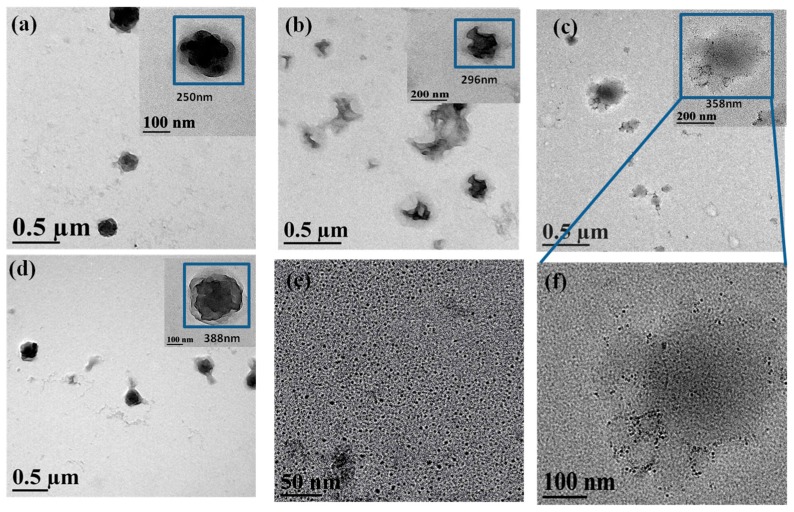
High-resolution transmission electron microscopy (HR-TEM) images of drug loaded MPEG-PDLLA/C60 nanocomposites prepared from different added amounts of C_60_ to the MPEG-PDLLA/C60 mixed solutions: (**a**) 0%; (**b**) 1%; (**c**) 5%; (**d**) 10%; (**e**) 100%; and (**f**) 5%.

The High-resolution transmission electron microscopy (HR-TEM) analysis were also performed to get further details into the MPEG-PDLLA/C60 nanocomposite structure and more directly study the participation of C60 in the formation process of the micelles. Figure 3 shows the HR-TEM images of the MPEG-PDLLA/C60 nanocomposites having C60 added amount of 0, 1, 5, and 10 wt%, respectively. The morphology of nanocomposites observed in HR-TEM images appeared to significant difference compared with that observed in the corresponding TEM images. The MPEG-PDLLA block copolymer micelle without C60 added exhibited spherical structures characterized by some pronounced protrusions on their surface (cf. Figure 3a). Moreover, a closer inspection of the HR-TEM image reveals that the periphery of the spherical structures is surrounded by a circle of light colored shadow. When a small amount of C60 (1 wt%) was added, the obtained MPEG-PDLLA/C60 nanocomposite structure showed an irregular aggregated structure that located in the core of a circular light colored shadow as indicated in Figure 3b. At an addition of C60 (5 wt%), we found that the morphology of MPEG-PDLLA/C60 nanocomposite structure liked a thin fried egg in the HR-TEM images as shown in Figure 3c. Closely observed the HR-TEM image, the C60 could be well-recognized and dispersed within the MPEG-PDLLA/C60 nanocomposite structure (cf. Figure 3f). Furthermore, the recognized C60 in the nanocomposites have similar size with that the pure C60 as indicated in Figure 3e. When the C60 added amounts reached 10 wt% the lamellar sphere structure was observed in the HR-TEM images, as shown in Figure 3d.

It is generally accepted that the electron beam used in a transmission electron microscope usually causes damage to specimen, especially to the biodegradable polymer, and leads to the temporary or permanent change in the chemical and physical properties of specimens [32,33]. As MPEG-PDLLA specimens is an insulator, its non-conducting nature can cause both charge and thermal build-up in the sample. Thus, the MPEG-PDLLA specimen is susceptible to melting or thermal degradation when observed by HR-TEM at high magnitude and a high accelerating voltages operating condition. The local melting or thermal degradation first occurred to the surface of the sample and could lead to the morphology of MPEG-PDLLA block copolymer micelles changing, as appeared in Figure 3a. The shadow surrounded the spherical structures may be some thermal decomposition production of MPEG-PDLLA block copolymer. C60 have good thermal conductivity and are also very easily excited to generate singlet oxygen. When the HR-TEM test was carried out on the MPEG-PDLLA/C60 nanocomposite specimens, the absorption coefficient of specimens for the incident radiation was improved due to the addition of C60 and the increase in thermal conductivity also increases thermal conduction from the specimen surface to the interior of specimen. Thereby, the vicinity of C60 located in the nanocomposites would get rapidly heated and its temperature could become high enough to initiate thermal degradation of MPEG-PDLLA. In addition, the heated C60 may also be excited to generate singlet oxygen within the MPEG-PDLLA/C60 nanocomposites that may further accelerate the degradation of MPEG-PDLLA. Therefore, When a small amount of C60 (1%) was added, the C60 molecules were only incorporated in part region within the nanocomposite and the PDLLA near the C60 was almost completely degraded since to the beam heat was concentrated in these vicinity of C60, as observed in Figure 3b, where some gaps existed in the edge of the aggregated structure. In the case of an addition of C60 increased to 5%, the more C60 molecules were dispersed homogeneously in the nanocomposites which help the heat was conducted evenly throughout the nanocomposite and, thus, led to the whole nanocomposite micelles to evenly degrade and collapse. In this case, the incorporated C60 in nanocomposite was also recognized in the HR-TEM image (cf. Figure 3f). However, when the C60 added amount reached 10 wt%, the degradation of MPEG-PDLLA/C60 nanocomposite seems to decrease instead. This phenomenon may be attributed to the increasing C60 additions resulted in the C60 aggregating or contacting with each other within the nanocomposites. In such a state, the area that heated by the C60 would increase and, thus, the local temperature rise of nanocomposites appeared to decrease. In addition, the singlet oxygen generated by C60 also easily quench each other in the C60 aggregated or contacted state. Therefore, the degradation degree of MPEG-PDLLA/C60 nanocomposites, instead appeared to decrease as the C60 added amounts increased to 10 wt%.

It is evident that the analysis of the structural change of MPEG-PDLLA/C60 nanocomposites associated with beam damage by HR-TEM provides substantial insight into the MPEG-PDLLA/C60 nanocomposite structure and the participation of C60 in the formation process of the micelles. However, the uncontrollable beam damage by the HR-TEM brings uncertainty to whether the explanation for the damage phenomena observed in HR-TEM images is reasonable.

**Figure 4:**
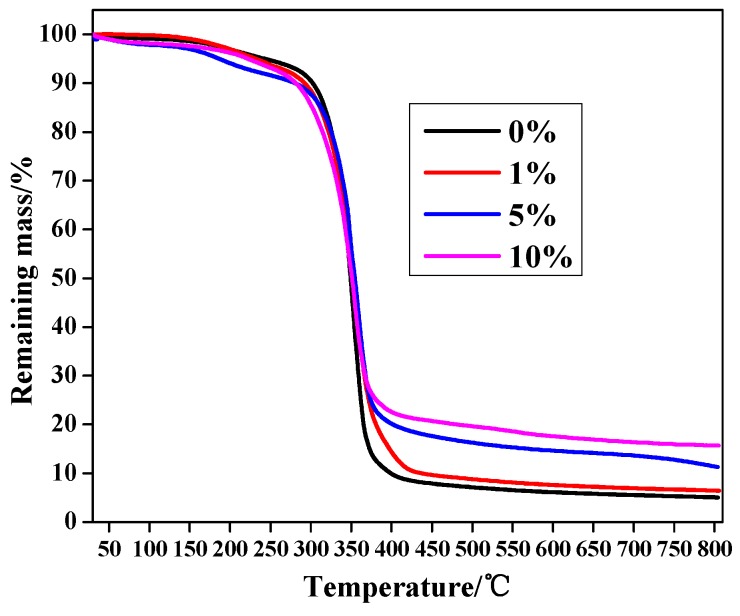
TGA curves (30–800 °C) of drug-loaded MPEG-PDLLA/C60 nanocomposites prepared from different added amounts of C60 to the C60/MPEG-PDLLA mixed solutions.

Thermal gravimetric analysis (TGA) was performed on the MPEG-PDLLA micelle and MPEG-PDLLA/C60 nanocomposites with the different C60 added amounts to more precisely determine the effect of the C60 addition on the nanocomposite structure and obtain additional confirmation of C60 content in the nanocomposites. The thermal decomposition profiles of the neat MPEG-PDLLA micelle and MPEG-PDLLA/C60 nanocomposites under a nitrogen atmosphere are given in Figure 4.

The experimental results showed that the weight loss of the MPEG-PDLLA/C60 nanocomposites exhibit different behaviors in the different temperature ranges. Interestingly, the MPEG-PDLLA/C60 nanocomposites having C60 added amounts of 5 wt% and 10 wt% began to gently decompose even at about 50 °C. The weight loss of nanocomposites in so early a stage should be attributed to the singlet oxygen generated by the heated C60 that catalyzed the thermal degradation of MPEG-PDLLA. In the range of 165–270 °C, all of the samples appeared to decompose and the addition of C60 increased the mass loss rate. However, the increasing mass loss rate shows different upon the C60 additions. The MPEG-PDLLA/C60 nanocomposites prepared by only a small added amount of C60 (1 wt%) show a silght increase in the mass loss rate compared to that of pure MPEG-PDLLA micelles. At an improved addition of C60 (5 wt%), the mass loss rate was obviously accelerated as indicated in Figure 4. However, further increase the addition of C60 to 10 wt%, the mass loss rate of the nanocomposites decreases close to that of the MPEG-PDLLA/C60 nanocomposites having a C60 added amount of 1 wt%.

It is obvious that the weight loss change of nanocomposites upon the C60 added amounts in the TGA curve is consistent with that of the damage degree of the nanocomposte structures observed in the HR-TEM images (cf. Figure 3). These TGA data verify our assumption that an small addition of C60 could accelerate the increase in local temperature of nanocomposite specimens and the heated C60 was also easily excited to generate the singlet oxygen that further promote the degradation of MPEG-PDLLA/C60 nanocomposites even at a low temperature; moreover, the weight loss rate of MPEG-PDLLA/C60 nanocomposites indeed decreased at a high C60 addition in the MPEG-PDLLA/C60 mixed solution. Please note, in this context, that the weight loss rate of MPEG-PDLLA/C60 nanocomposite with any added amount of C60 was always faster than that of pure MPEG-PDLLA micelles in the range of 165–270 °C, where the MPEG-PDLLA molecules did not appear to obviously degrade. Although the addition of C60 increased the initial mass loss rate compared to that of MPEG-PDLLA, the final residual mass of specimens after the nanocomposites degraded completely increases upon the increasing C60 additions. This increasing final remaining mass is attributed to the high thermal stability of the C60 and the increasing C60 content in the MPEG-PDLLA/C60 nanocomposite upon the increasing C60 additions.

**Figure 5:**
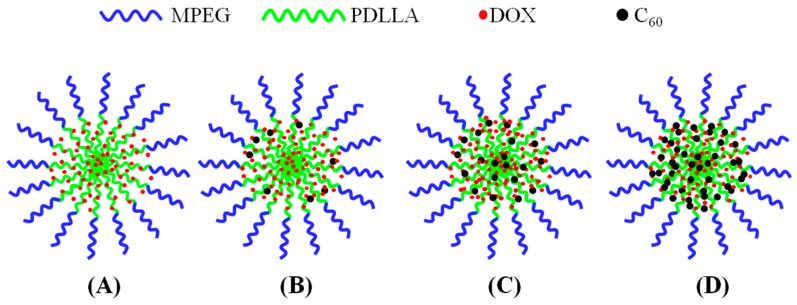
Schematic representation of the possible formation structures of MPEG-PDLLA/C60 nanocomposites prepared from different added amounts of C60 to the MPEG-PDLLA/C60 mixed solutions: no addition of C60 (**A**); a low addition of C60 (**B**,**C**); and a high addition of C60 (**D**).

In combination of the above results, a clearer picture on how C60 incorporated in MPEG-PDLLA/C60 nanocomposites could be was obtained as outlined in Figure 5.

To explore the possibility of using the MPEG-PDLLA/C60 nanocomposites as nanocarriers for chemotherapeutic drugs, the drug loading and drug release experiments were carried out. Table 2 shows the drug entrapment of the MPEG-PDLLA/C60 nanocomposites with different C60 additions. An increase in drug entrapment with the initially increasing additions of C60 was observed. However, after the addition of C60 reached 5 wt%, the drug entrapment decreased with further increases in the addition of C60. Considering that the drug entrapment of the pure C60 is 2.43 and less than that of all of the MPEG-PDLLA/C60 nanocomposites, the increase in DOX loading efficiency of MPEG-PDLLA/C60 nanocomposites is not caused by the interactions between C60 and DOX. A possible reason for the drug entrapment change upon the addition of C60 could be that the incorporation of the spherical C60 in the core of MPEG-PDLLA/C60 nanocomposites would lead to an increase in the molecular chain spacing of PDLLA segments in the vicinity of C60 and, thus, produce a larger cargo space to increase the drug entrapment as indicated in Figure 5B,C. When the C60 is added to a certain amount, although the size of MPEG-PDLLA/C60 nanocomposites was still increasing, the new produced cargo space for drug entrapment could be reduced due to the aggregation of C60 within the adjacent region of the nanocomposites as indicated Figure 5D.

**Figure 6:**
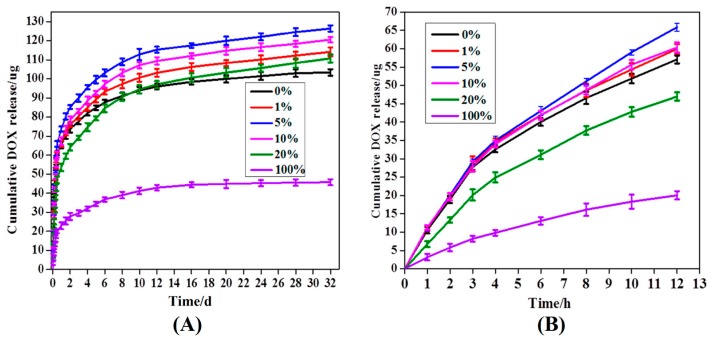
Release profiles of DOX from DOX-loaded of MPEG-PDLLA/C60 nanocomposites prepared from the different added amounts of C60 to the MPEG-PDLLA/C60 mixed solutions in 30 d (**A**) and 12 h (**B**).

The in vitro DOX release from the MPEG-PDLLA/C60 nanocomposites with different C60 additions was studied in PBS at 37 °C and pH 7.4. The release profile is presented with respect to a cumulative release versus time as shown in Figure 6. For all nanocomposites, the DOX release amounts are characterized by an initial burst followed by a steady-release phase. At the initial burst release stage, the amounts of DOX released from the MPEG-PDLLA/C60 nanocomposites with different C60 contents were found to be almost identical, except the 20 wt% C60 additions (cf. Figure 6B). However, after the first burst release stage, the released amount of DOX exhibits differently upon the additions of C60 at the steady-release stage. When 1 wt% of C60 was added to the MPEG-PDLLA, the released amount of DOX from the nanocompostes was found to be faster than that from the pure MPEG-PDLLA micelles or pure C60. The released amount of DOX was further obviously improved upon addition of 5 wt% C60 to the MPEG-PDLLA. However, when the C60 added amount was raised to 10 wt% in the MPEG-PDLLA/C60 mixed solution, the DOX-loaded nanocomposites showed a decreasing DOX release. Moreover, the released amount of DOX from the nanocomposites decreased obviously upon further increasing C60 additions to 20% in the MPEG-PDLLA/C60 mixed solution, and even less than that of the pure MPEG-PDLLA micelles in 10 days. Moreover, the amounts of DOX released from the MPEG-PDLLA/C60 nanocomposites with 20 wt% C60 addition is even less than that of the pure MPEG-PDLLA micelles in eight days. Please note, in this context, that the amount of DOX released from the MPEG-PDLLA/C60 mixed micelles with any added amount of C60 was always faster than that of pure MPEG-PDLLA micelles or C60 except the 20 wt% C60 additions.

The rate of drug released from micelle is closely related with its localization within the micelle. Generally, the initial burst release of DOX can be attributed to the DOX molecules adsorbed within the shell or the interface between the core and shell of nanocomposite micelle [19]. In this situation, the release of drug molecules that located at the core-shell interface or in the out shell was very fast and not influenced by the physical-chemical properties of core of a micelle [34]. Thus, the effect of C60 incorporated in the core of MPEG-PDLLA micelles on drug release was not apparent at the burst release stage. At the steady-release stage, the release profile features a slow release phase governed by a slow DOX diffusion from the hydrophobic core of the micelles. In this stage, the interactions between the drug and the core-forming block and the physical state of the micelle core both have apparent effect on the drug release [20,35]. When C60 was incorporated in the rigid core of MPEG-PDLLA micelle by the hydrophobic interactions, the molecular chain space of PDLLA segments in the vicinity of C60 increased and, thus, produced a larger cargo space to increase the drug entrapment as discussed in drug entrapment section while the interaction between the DOX and the PDLLA segment was weakened in this region. The expanded space and the weakened interaction, thus, help accelerate the hydrophobic drug release from the core as highlighted in Figure 5. These two factors are also responsible for the amount of DOX released from the MPEG-PDLLA/C60 nanocomposites with any added amount of C60 was always faster than that of pure MPEG-PDLLA micelles or C60, except the 20 wt% C60 additions. Such influence increases upon the increasing C60 addition in the MPEG-PDLLA/C60 mixed solution as discussed above. Therefore, the increase of the DOX released amount from the MPEG-PDLLA/C60 nanocomposites upon addition of C60 was observed. However, when the amount of C60 is added to a certain amount, although the factor of weakened interaction between the DOX molecules with PDLLA chain segment still remains, the expanded space within the nanocomposites may decrease by the overloaded C60 and these C60 also produced a steric hindrance for DOX released from the nanocomposites as highlighted in Figure 5D. Therefore, the further addition of C60 caused an obvious decrease in the drug release when the C60 added in the MPEG-PDLLA/C60 mixed solution reached a concentration above 5 wt%, and even the occurrence of the drug release rate of nanocomposites prepared from 20 wt% C60 addition is slower than that of pure MPEG-PDLLA micelles during the first eight days.

## 3. Materials and Methods

### 3.1. Materials

Monomethoxy poly(ethylene glycol)-block-poly(d,l-lactide) (MPEG-PDLLA) (MPEG, Mw = 2000, PDLLA, Mw = 10,000) was purchased from Jinan Daigang Biological Technology Company (Jinan, China). C60 was purchased from Nanjing Xianfeng Nano-Material Technology Company (Nanjing, China). Doxorubicin hydrochloride (DOX·HCl) was purchased from Beijing Huafeng United Technology Company (Beijing, China). Dimethyl sulfoxide (DMSO) was purchased from Sinophaim Chemical Reagent Company (Shanghai, China). Phosphate buffer powder (PBS) and dialysis tube (molecular weight cut off = 8000–12,000) were purchased from Shanghai Yuanye Biological Technology Company (Shanghai, China). All other reagents were of analytical grade and were used as received.

### 3.2. Methods

#### 3.2.1. Preparation of the MPEG-PDLLA/C60 Nanocomposites

The MPEG-PDLLA/C60 nanocomposites were prepared via a membrane dialysis method. In a general procedure, DOX·HCl (10 mg) was neutralized with 1 mL excess triethylamine in 5 mL of DMSO. The diblock copolymer MPEG-PDLLA (40 mg) and a given amount of C60 were then added to the DOX solution (the addition amount of C60 to MPEG-PDLLA was 0%, 1%, 5%, 10% and 20%, respectively). After 2 h of stirring at room temperature, the mixed solution was dialyzed against deionized water with a dialysis tube (molecular weight cut off = 8000–12,000). MPEG-PDLLA/C60 nanocomposites formed as indicated by the appearance of opalescence in the solution. The nanocomposites were further purified by continual dialysis against deionized water for three days and the deionized water was replaced every 6 h during this time period.

#### 3.2.2. Characterization of MPEG-PDLLA/C60 Nanocomposites

The morphology of the samples was analyzed using a transmission electron microscope (TEM) with a JEOL JEM-2010 microscope operating at an accelerating voltage of 100 KV. A sample was prepared by placing a freshly-prepared MPEG-PDLLA/C60 nanocomposite solution onto a carbon-coated copper grid forming a thin liquid film; the sample was then air-dried at room temperature. An average particle size could be calculated from the TEM images by averaging the size of more than 100 micelles. High-resolution transmission electron microscopy (HR-TEM) was carried out using a JEM-2100F high-resolution transmission electron microscope operated at 200 KV

The particle size and size distribution of the nanocomposite were measured using dynamic light scattering (DLS). DLS studies were conducted using a Zetasizer Nano ZS90 instrument (Malvern Instruments) equipped with a multipurpose autotitrator (MPT-2) at a fixed scattering angle of 90°. The data were processed by cumulative analysis of the experimental correlation function, and the particle diameters were calculated from the computed diffusion coefficients using the Stokes-Einstein equation. Each reported measurement was conducted for three total runs.

Thermal gravimetric analyses (TGA) of MPEG-PDLLA/C60 nanocomposites were carried out using a NETZSCH STA 449C instrument at 10 °C/min from 30 to 800 °C in nitrogen (flow rate of 60 cm^3^/min) for the original nanocomposite samples (about 8 mg) in a platinum pan.

To determine the corresponding DOX loading level of the MPEG-PDLLA/C60 nanocomposites, a known amount of DOX-loaded nanocomposites was dissolved in 1 mL of DMSO. The DOX concentration was measured by a UV-VIS SP-752 PC spectrophotometer using an absorbance wavelength of 480 nm. The drug entrapment (DE %) was calculated according to the following equation:

$$\text{DE}\%=\frac{\mathrm{weight}\;\mathrm{of}\;\mathrm{DOX}\;\mathrm{loaded}\;\mathrm{in}\;\mathrm{nanocomposites}}{\mathrm{weight}\;\mathrm{of}\;\mathrm{drug}\;\mathrm{carriers}}\times 100\%$$

#### 3.2.3. In Vitro Drug Release

A 2.0 mL aqueous dispersion of drug loaded MPEG-PDLLA/C60 nanocomposites (2.0 g/L) was transferred to a dialysis tube with a molecular weight cutoff of 8000–12,000 and was then immersed in 5 mL of PBS buffer solution (pH 7.4, 10 mM), thermostatted at 37 °C, and shaken at a speed of 150 rpm for 30 days. Five milliliters of the PBS buffer solution was removed at different time intervals and was replaced with an equal volume of fresh release medium. The concentration of DOX in the samples was determined by UV absorbance at 480 nm. A series of parallel experiments was conducted at varying amounts of C60.

## 4. Conclusions

In conclusion, the MPEG-PDLLA/C60 nanocomposites were explored as nanocarriers for hydrophobic drug DOX to combine the advantages of both amphiphilic block copolymer and C60 delivery systems. The effects of adding C60 to methoxy polyethylene glycol-poly(d,l-lactic acid) (MPEG-PDLLA) micelles on the structure and corresponding drug release was investigated. The incorporation of the spherical C60 in the core of MPEG-PDLLA/C60 nanocomposites led to an increase in the molecular chain space of PDLLA segments in the vicinity of C60 and, thus, producing a larger cargo space to increase the drug entrapment and accelerate the drug release. However, a sufficient addition of C60 may result in an aggregation of C60 within the nanocomposite that decreased the drug entrapment and produced a steric hindrance for DOX released from the nanocomposites. These findings successfully validated the strategy of using C60 as an easy and efficient drug release regulator for amphiphilic block copolymer nano-micelles. It is noted that the unique photochemical property of C60 easily generates singlet oxygen and also provides a potential for the MPEG-PDLLA/C60 nanocomposites used as nanocarriers for the combined chemotherapy and photodynamic therapy. Further experimental studies to substantiate this latter notion will be carried out in our laboratory.

## Figures and Tables

**Table 1:** Size distributions of drug-loaded MPEG-PDLLA/C60 nanocomposites determined by DLS and TEM.

C60 Addition Amount	0%	1%	5%	10%	20%	100%
DLS diameter (nm)	280	301	322	338	384	-
TEM diameter (nm)	231	254	265	286	318	4

**Table 2:** Drug-entrapment (DE %) of MPEG-PDLLA/C60 nanocomposites prepared from the MPEG-PDLLA/C60 mixed solutions with different C60 additions.

C60 Addition Amount	0%	1%	5%	10%	20%	100%
Drug-entrapment (DE %)	4.32	4.99	6.22	5.68	5.81	2.43
